# Proteomic Analysis of Salt-Responsive Proteins in the Leaves of Mangrove *Kandelia candel* during Short-Term Stress

**DOI:** 10.1371/journal.pone.0083141

**Published:** 2014-01-08

**Authors:** Lingxia Wang, Xiao Liu, Meng Liang, Fanglin Tan, Wenyu Liang, Yiyong Chen, Yongxiang Lin, Li Huang, Jianhong Xing, Wei Chen

**Affiliations:** 1 Key Laboratory of Ministry of Education for Genetics, Breeding and Multiple Utilization of Crops, Fujian Agriculture and Forestry University, Fuzhou, Fujian, China; 2 School of Life Sciences, Fujian Agriculture and Forestry University, Fuzhou, Fujian, China; 3 Fujian Academy of Forestry, Fuzhou, Fujian, China; 4 School of Life Sciences, Ningxia University, Yinchuan, Ningxia, China; University of Louisville, United States of America

## Abstract

Salt stress is a major abiotic stress that limits crop productivity in many regions of the world. A comparative proteomic approach to identify salt stress-responsive proteins and to understand the molecular mechanisms was carried out in the woody halophyte *Kandelia candel*. Four-leaf-old *K. candel* seedlings were exposed to 150 (control), 300, 450, and 600 mM NaCl for 3 days. Proteins extracted from the leaves of *K. candel* seedlings were separated by two-dimensional gel electrophoresis (2-DE). More than 900 protein spots were detected on each gel, and 53 differentially expressed protein spots were located with at least two-fold differences in abundance on 2-DE maps, of which 48 were identified by matrix-assisted laser desorption ionization time-of-flight/time-of-flight mass spectrometry (MALDI-TOF-TOF/MS). The results showed that *K. candel* could withstand up to 450 mM NaCl stress by up-regulating proteins that are mainly involved in photosynthesis, respiration and energy metabolism, Na^+^ compartmentalization, protein folding and assembly, and signal transduction. Physiological data, including superoxide dismutase (SOD) and dehydroascorbate reductase (DHAR) activities, hydrogen peroxide (H_2_O_2_) and superoxide anion radicals (O_2_^−^) contents, as well as Na^+^ content and K^+^/Na^+^ ratios all correlated well with our proteomic results. This study provides new global insights into woody halophyte salt stress responses. Identification of differentially expressed proteins promotes better understanding of the molecular basis for salt stress reduction in *K. candel*.

## Introduction

Salinity is one of the major abiotic stresses that pose a severe threat to agricultural productivity in many global regions [1]. Salinity can cause a variety of changes in the metabolism of higher plants, such as photosynthetic and respiratory repression, osmotic stress, ion toxicity, oxidative stress, and nutrient deficiencies, leading to a decrease in the growth and productivity of plants [2]–[9].

Mangroves, which are intertidal plants that form unique communities along tropical and subtropical coastal areas, constitute a model for salt-tolerant xylophytes [10], [11]. Several mangrove tree species display optimal growth in the presence of moderate salt stress conditions, whereas severe salinity restricts their growth [12]. The most striking feature of mangroves is their ability to tolerate salt up to seawater concentrations (∼500 mM NaCl); this has attracted the attention of many scientists interested in elucidating this phenomenon [13], [14]. Previous studies indicated that mangroves have evolved diverse strategies to respond to high salinity in their environment. Some species actively excrete excess salt by means of specialized salt glands in their leaves, while other species excrete salt by ultrafiltration at the root cell membranes of cortical cells [11]. Hotta et al. [15] reported that the response mechanism of mangroves to salt may be linked to changes in vacuolar size. Moreover, accumulation of compatible solutes and induction of antioxidative enzymes constitute other biochemical mechanisms of mangroves against high salinity [13]. Some reports studied the effect on photosynthetic parameters such as the rate of photosynthesis, stomatal conductance [12], [16], and chloroplast structure and function [17]. In addition, some studies investigated the changes in gene expressions under salt stress [18]–[21]. Tanaka et al. [22] reported that the gene of vacuolar Na^+^/H^+^ antiporter from *Bruguiera sexangula* played an important role in cellular salinity adjustments. Miyama et al. [23] identified 14,842 expressed sequence tags from leaves and roots of *B. gymnorrhiza* under high salinity or hormone treatments. The mRNA expressions of Cu–Zn SOD, catalase and ferritin in response to salt stress in *A. marina* were studied and their role in oxidative stress response was confirmed [24]. The transcriptional response to high salinity and hyperosmotic stress in *B. gymnorrhiza* was investigated using the microarray approach. Eight hundred sixty-five genes showed significant differential expression under salt and osmotic stress [25]. Proteomic responses to salinity have been studied in various herbaceous plants including rice [1], [26]–[28], canola [4], soybean [29], wheat [30], barley [31], the model plant *Arabidopsis thaliana* [32], [33], cucumber [34], and the facultative model halophyte *Salicornia europaea* [35]. Understanding how different levels of protein abundance form the basis of salt tolerance mechanisms in mangroves will shed new light and give a new dimension to salt stress research. To date, few studies have been dedicated to observing the effects of salt stress on salt-tolerant xylophytes. However, two studies reported effects of long term salt stress (12 d and 45 d, respectively) on the abundance of proteins in mangrove, *B. gymnorrhiza* (L.) Lam [36], [37]. Tada and Kashimura [36] reported that fructose-1,6-bisphosphate (FBP) aldolase and osmotin were found related to salt tolerance in *B. gymnorrhiza* roots. Zhu et al. [37] reported that photosynthesis-related proteins and antioxidant enzymes in the leaves were up-regulated by 200 mM NaCl and down-regulated by 500 mM NaCl suggesting that the salt-responsive mechanisms in *B. gymnorrhiza* at these NaCl concentrations were different.

*K. candel* is one of the most important species of mangrove around the coasts of South and Southeast Asia from western India to Borneo. It has developed various ways to adapt and respond to changes in concentrations of salinity in the environment. However, to our knowledge, no information is available describing how *K. candel* responds to short-term (3 days) exposure to NaCl at the level of protein expression. In the present study, proteome analysis was used to identify the mechanisms responsible for the response of *K. candel* to salt stress. The objective of the current study is to characterize specific metabolic and regulatory mechanisms of salt tolerance in *K. candel*. These proteomic data contribute significantly to our understanding of protein alteration in this salt-tolerant xylophyta. The results give insights into the molecular mechanism in responses to salinity stress in *K. candel* seedlings.

## Results

### Changes in SOD and DHAR activities and H_2_O_2_ and O_2_^−^ contents

**Figure 1:**
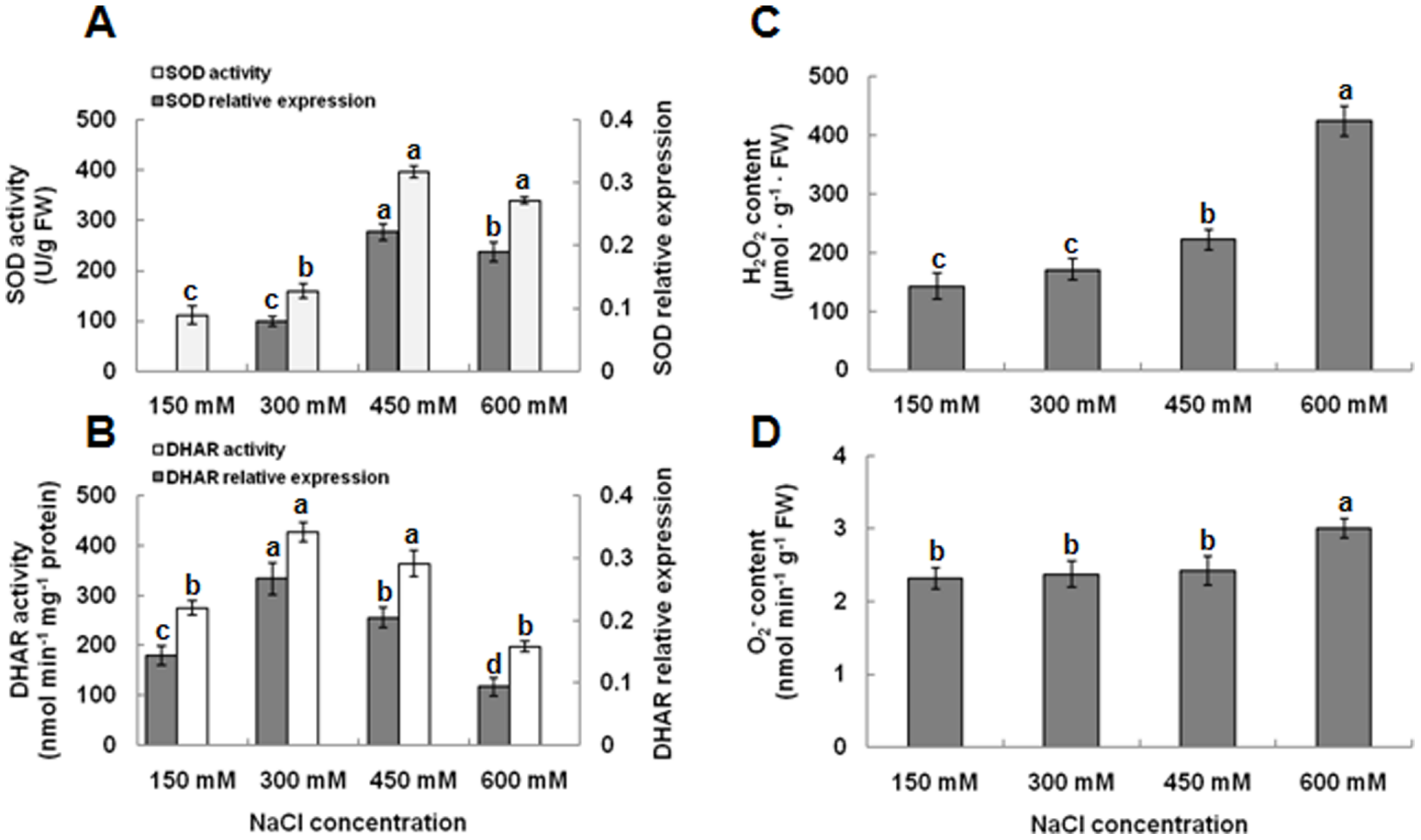
Effect of salinity on the activites of SOD and DHAR and contents of H_2_O_2_ and O_2_^−^ in *K. candel* leaves. Values (means ± SD) were determined from three independent experiments (n = 3) after plants had been treated with 150, 300, 450, and 600 mM NaCl for 3 d.

Induction of antioxidant enzymes is an important protective mechanism for minimizing oxidative damage from salt stress. Analysis of SOD indicated significant changes in activity upon salt treatment. The SOD activity peaked in response to 450 mM NaCl (356% of the control) (Figure 1A), whereas the DHAR activity peaked at 300 mM (155% of the control) (Figure 1B). Furthermore, at 600 mM NaCl, the SOD levels were still significantly higher than the control (150 mM) value, whereas, with DHAR (Figure 1B), the 600 mM value is not significantly different from the 150 mM activity. High salinity concentration leads to the production of large amounts of H_2_O_2_ and O_2_^−^. An increase in the content of H_2_O_2_ was detected with increasing salinity levels (Figure 1C). H_2_O_2_ content reached up to 198% at 600 mM NaCl when compared with the control (Figure 1C). However, O_2_^−^ contents obviously increased only by 25% under 600 mM salt treatment, but no significantly different between 450 mM and 600 mM salt-treated seedling leaves (Figure 1D).

### Na^+^ and K^+^ ion content analysis

**Figure 2:**
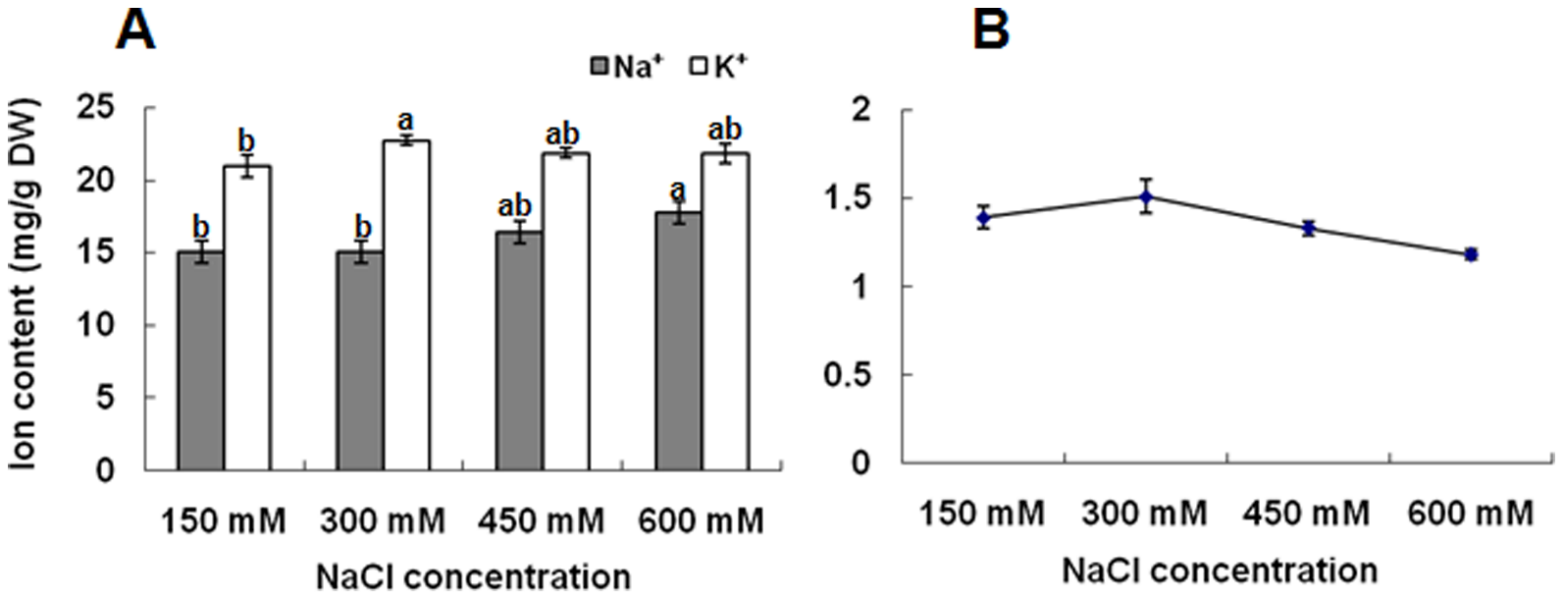
Effect of salinity on ionic contents in *K. candel* leaves. (A) Na^+^ and K^+^ concentration in leaves; (B) K^+^/Na^+^ ratio in leaves. Values (means ± SD) were determined from five independent experiments (n = 5) after plants had been treated with 150, 300, 450, and 600 mM NaCl for 3 d.

Na^+^ concentrations in *K. candel* leaves were affected by salinity. Compared with the control plants, the Na^+^ concentration in leaves increased slightly after salt treatment for 3 d (Figure 2A). Salinity did not significantly affect the K^+^ content in *K. candel* leaves under salt treatments in this study (Figure 2A). Additionally, K^+^/Na^+^ ratios only decreased slightly with increasing salt content (Figure 2B).

### Proteomic and hierarchical clustering analysis

**Figure 3:**
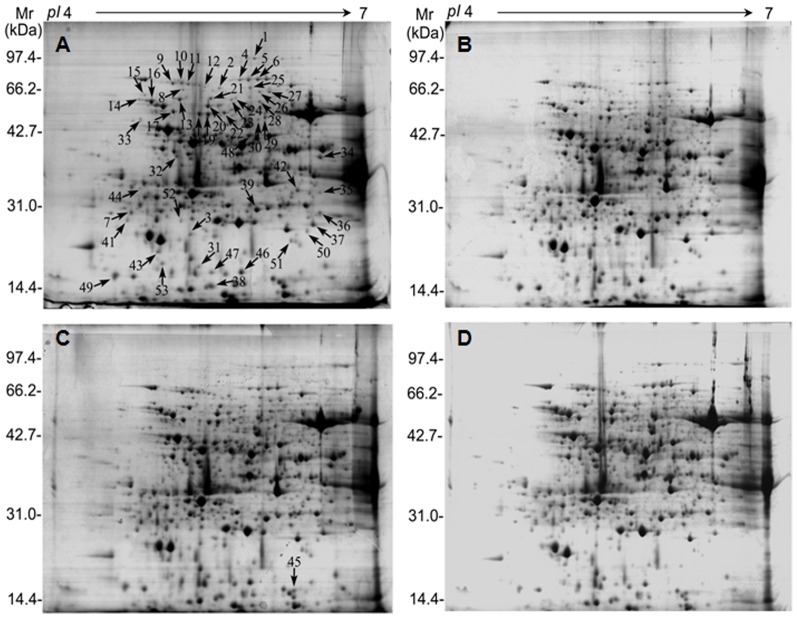
Representative 2-DE gel images of *K. candel* leaves treated with NaCl. An equal amount (1.5 mg) of total proteins was loaded on each gel strip (pH 4–7). After IEF, 12.5% SDS-PAGE gels were used for second dimension separation. Protein spots were visualized using CBB staining. The 2-DE maps of proteins from leaves of the control samples (150 mM NaCl) and the NaCl-treated samples (300, 450, and 600 mM) are presented. Proteins differentially regulated in response to salinity are numbered in pairs of control and NaCl-treated maps. Arrows indicate the 48 spots that showed significant changes in the control and NaCl-treated samples. Identification of these protein spots by MALDI-TOF-TOF-MS/MS is marked with arrows in (A) and given in Table 1.

**Figure 4:**
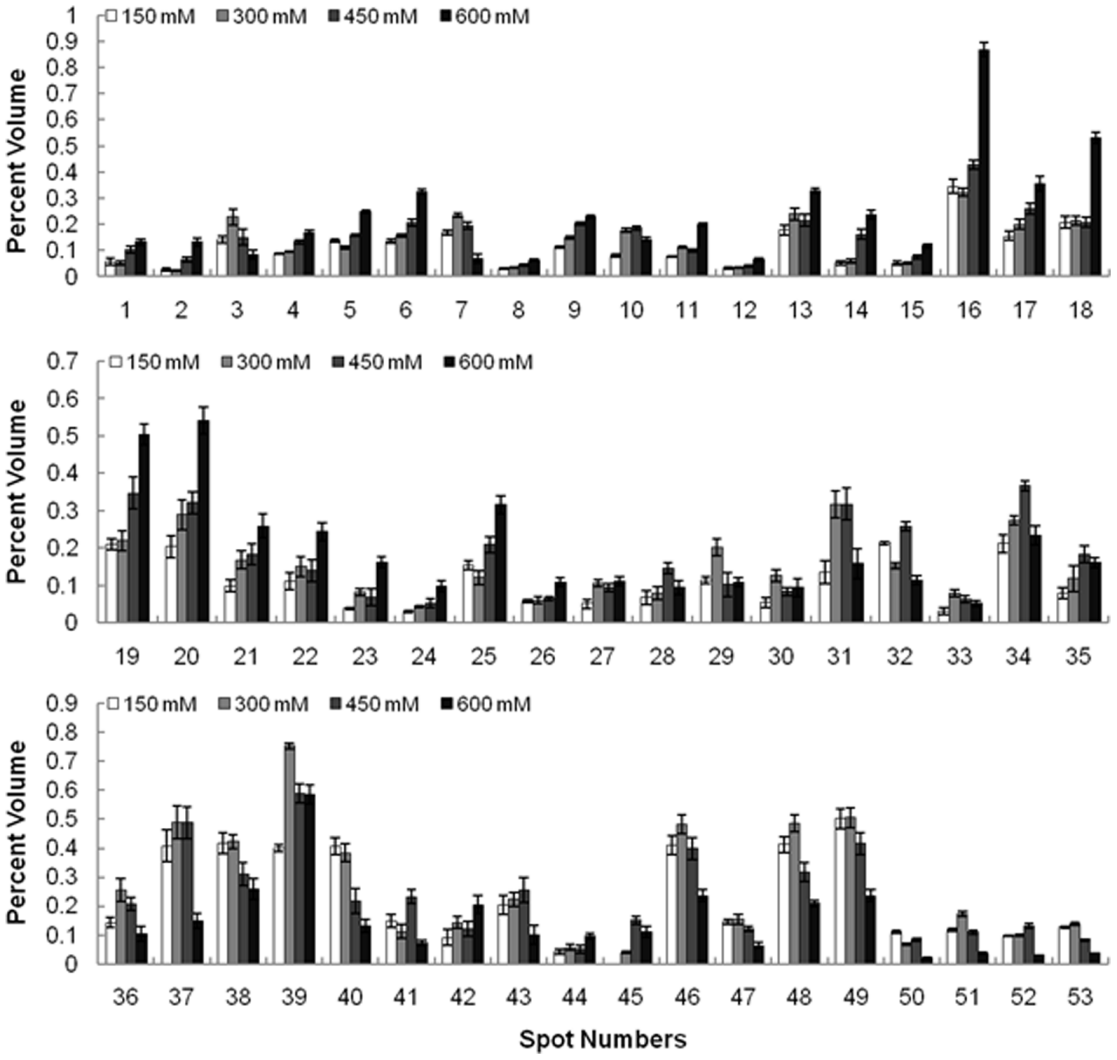
Relative expression levels of 53 differentially expressed proteins of *K. candel* leaves under salt stress. Changes in protein expression under salt stress were calculated by Image Master software 5.0. Mean of relative protein abundance and standard error. Three treatments including control (150, 300, 450, 600 mM NaCl for 72 h) were performed.

**Figure 5:**
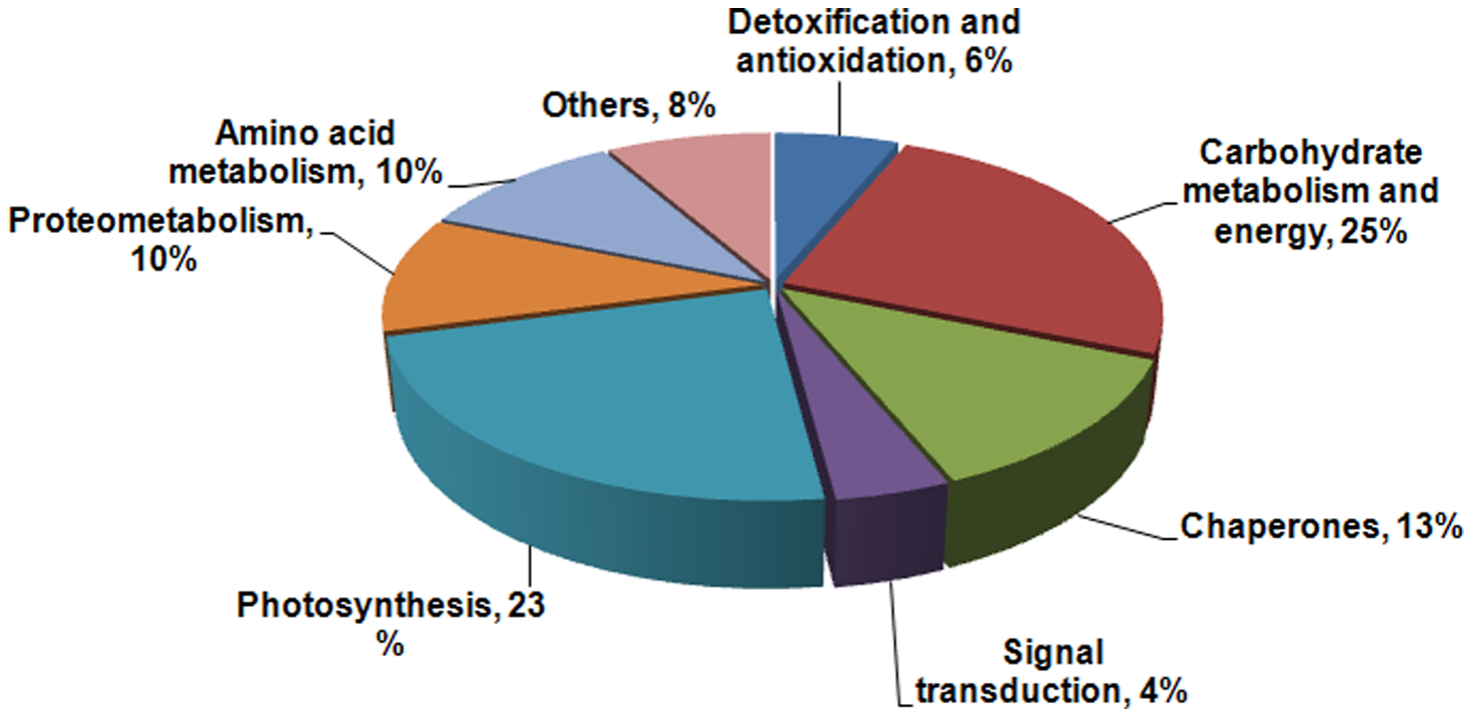
Functional classification of differentially expressed proteins identified in the seedling leaves of *K. candel* under salt stress. The classification is based on KEGG (http://www.kegg.jp/kegg/pathway.html) and previous literature.

Proteins extracted from *K. candel* leaves following NaCl treatment were separated by 2-DE. More than 900 protein spots were detected in each gel by ImageMaster software (Figure 3). Fifty-three differentially expressed protein spots showed two-fold increases in abundance. MS analysis of the protein spots resulted in the identification of 48 proteins. The identified proteins are listed in Table 1 and Figure 4. In this study, all identified proteins were categorized into eight functional groups using the MapMan ontology as shown in Figure 5.

**Table 1:** Protein identities and their relative changes under salt stress in *Kandelia candel* leaves from 2D-Gel analysis.

Spot No^a^	Protein Name	Species	Accession No^b^	Matched peptide sequences (m/z)^c^	Theor.^d^ MW(kDa)/p*I*	Exp.^d^ MW(kDa)/p*I*	PS/IS^e^	MP/TP^f^	Cov%^g^
**Photosynthesis**									
13	RuBisCO large subunit-binding protein subunit beta, chloroplast precursor (60 kDa chaperonin subunit beta) (CPN-60 beta)	*Pisum sativum*	P08927	DLINILEDAIR(1284.7329) SQYLDDIAILTGGTVIR(1835.0303)	62.95/5.85	59.15/5.34	214/135	2/20	34%
7	Chlorophyll a–b binding protein 2,chloroplastic	*Populus euphratica*	P84990	NRELEVIHSR(1252.7225) SAPQSIWYGPDRPK(1601.8837)	37.99/8.2	27.02/4.89	158/116	2/4	100%
14	rubisco subunit binding-protein alpha subunit, ruba, putative	*Ricinus communis*	XP_002534347	LADAVGLTLGPR(1182.682)	53.17/5.25	58.4/4.95	99/79	1/8	13%
16	Rubisco subunit binding-protein alpha subunit, ruba, putative	*Ricinus communis*	XP_002534347	LADAVGLTLGPR(1182.6986)	53.17/5.25	57.58/5.08	139/95	1/11	23%
31	ribulose-1,5-bisphosphate carboxylase/oxygenase large subunit	*Kandelia candel*	AAB66915	ALRLEDLR(985.5867) DTDILSAFR(1037.5345) LEDLRIPPAYSK(1401.792) TFQGPPHGIQVER(1465.7908) LTYYTPDYETKDTDILSAFR(2412.2302)	48.07/6.34	18.38/5.51	419/327	5/8	20%
35	ribulose-1,5-bisphosphate carboxylase/oxygenase large subunit	*Kandelia candel*	AAB66915	DNGLLLHIHR(1187.6913) EITAGFVDLLR(1233.7106) TFQGPPHGIQVER(1465.7932)	48.07/6.34	31.00/6.66	212/184	3/12	13%
37	chlorophyll A/B binding protein, putative	*Ricinus communis*	XP_002524566	QYFLGLEK(997.5484) WLAYGEIINGR(1291.7159)	29.36/6.85	25.69/6.61	149/130	2/5	18%
43	Oxygen evolving enhancer protein 2	*Bruguiera Gymnorhiza*	BAA96364	EVEFPGQVLR(1173.6053) QYYFLSVLTR(1289.6636)	17.53/4.91	20.82/5.14	107/94	2/3	18%
4	plastid transketolase	*Nicotiana tabacum*	ACF60500	TPSILALSR(957.5609) NPYWFNR(996.4607)	80.05/6.16	74.56/5.90	120/84	2/12	17%
5	plastid transketolase	*Nicotiana tabacum*	ACF60500	TPSILALSR(957.5534) NPYWFNR(996.4529) ALPTYTPESPADATR(1589.7661)	80.05/6.16	74.56/5.98	152/115	3/12	15%
6	transketolase 1	*Capsicum annuum*	CAA75777	NPYWFNR(996.4579) ALPTYTPESPADATR(1589.7644)	80.05/6.16	74.56/6.06	154/128	2/12	13%
**Carbohydrate metabolism and energy**									
1	aconitate hydratase 3	*Citrus clementina*	CBE71057	QVEIPFKPAR(1184.6525) INPLVPVDLVVDHSVQVDVAR (2284.2341)	98.04/5.89	98.1/6.04	181/165	2/11	10%
17	enolase	*Zea mays*	ACG31732	AAVPSGASTGVYEALELRDGGSDYLGK (2683.3953)	47.9/5.67	52.88/5.28	111/85	1/6	17%
18	mitochondrial F1-ATPase beta subunit	*Dimocarpus longan*	ACJ06633	VVDLLAPYQR(1173.6497) AHGGFSVFAGVGER(1390.6788) VGLTGLTVAEHFR(1399.7584) FTQANSEVSALLGR(1492.7637) IPSAVGYQPTLSTDLGGLQER (2202.1514)	59.81/6.18	52.5/5.51	571/520	5/12	27%
19	mitochondrial F1-ATPase beta subunit	*Dimocarpus longan*	ACJ06633	VVDLLAPYQR(1173.6666) AHGGFSVFAGVGER(1390.7012) VGLTGLTVAEHFR(1399.7795) FTQANSEVSALLGR(1492.7859) IPSAVGYQPTLSTDLGGLQER (2202.1875)	59.81/6.18	51.31/5.58	554/503	5/12	24%
20	ATP synthase CF1 alpha subunit	*Citrus sinensis*	YP_740460	ERIEQYNR(1107.5598) LIESPAPGIISR(1252.7411) IAQIPVSEAYLGR(1416.808) EAYPGDVFYLHSR(1553.7662) IVNIGTVLQVGDGIAR(1624.9561) VINALAKPIDGRGEISASESR (2183.219)	55.45/5.09	56.8/5.58	606/429	6/25	35%
22	F1 ATPase	*Pisum sativum*	BAA20135	VGLTGLTVAEHFR(1399.7831)	60.11/6.63	52.5/5.75	106/54	1/13	23%
25	succinate dehydrogenase, putative	*Ricinus communis*	XP_002530482	AASTILATGGYGR(1237.6605) LGANSLLDIVVFGR(1473.856) AVIELENYGLPFSR(1607.8613)	68.46/6.18	66.15/6.00	262/241	3/8	15%
26	Os01g0817700 (Putative phosphoglycerate mutase)	*Oryza sativa* Japonica Group	NP_001044625	VHILTDGR(910.5146) FGHVTFFWNGNR(1481.7295) LPSHYLVSPPEIER(1636.8873)	60.75/5.42	63.07/6.11	237/219	3/9	15%
27	2,3-bisphosphoglycerate independent phosphoglycerate mutase	*Ricinus communis*	XP_002519975	FGHVTFFWNGNR(1481.7295) LPSHYLVSPPEIER(1636.8873)	61.15/5.52	59.96/5.81	217/163	2/15	18%
32	Fructokinase-1	*Oryza sativa Indica Group*	A2WXV8	TALAFVTLR(991.6175) APGGAPANVAIAVAR(1334.7853)	34.71/5.14	36.98/5.31	186/142	2/9	32%
34	Malate dehydrogenase, cytoplasmic	*Mesembryantheum crystallinum*	O24047	NVIIWGNHSSTQYPDVNHATVK (2480.1733)	35.48/6.00	37.02/6.41	77/38	1/3	15%
39	triosephosphate isomerase	*Gossypium hirsutum*	ACJ11723	FFVGGNWK(954.5039)	27.19/6.00	25.33/6.04	85/48	1/7	35%
45	chloroplast superoxide dismutase	*Bruguiera gymnorhiza*	CAM98444	AFVVHELEDDLGKGGHELSLTTGNAGGR (2879.5146)	23.21/6.29	15.51/6.29	183/163	1/3	22%
**Detoxification and antioxidation**									
36	Dehydroascorbate reductase 1	*Actinidia deliciosa*	ADB85570	EYVIAGWEPK(1191.5714)	23.80/5.17	27.46/6.65	76/61	1/4	18%
2	vacuolar H^+^-ATPase	*Malus×domestica*	ABO33173	LHEDLTSGFR(1174.604) LAADTPLLTGQR(1255.723) VGHDNLIGEIIR(1335.7715) EASIYTGITIAEYFR(1733.9414) LTTFEDSEKESEYGYVR(2053.0273)	68.88/5.37	66.2/5.70	586/407	5/28	47%
**Chaperones**									
9	non-cell-autonomous heat shock cognate protein 70	*Cucurbita maxima*	AAN86274	NALENYAYNMR(1358.6045) STVHDVVLVGGSTR(1426.7528) TTPSYVAFTDSER(1473.6743) ARFEELNMDLFR(1540.7422) NAVVTVPAYFNDSQR(1680.8284) EQVFSTYSDNQPGVLIQVYEGER (2658.294)	70.77/5.13	70.88/5.28	672/545	6/23	42%
10	High molecular weight heat shock protein	*Malus×domestica*	AAF34134	NALENYAYNMR(1358.632) TTPSYVAFTDTER(1487.7235) NAVVTVPAYFNDSQR(1680.8619) EQVFSTYSDNQPGVLIQVYEGER (2658.268)	71.17/5.17	72.44/5.35	440/332	4/21	40%
11	Hsc70	*Solanum lycopersicum*	AAB42159	NALENYAYNMR(1358.632) TTPSYVAFTDTER(1487.7235)	71.47/5.18	70.88/5.42	429/332	4/19	29%
				NAVVTVPAYFNDSQR(1680.8619) EQVFSTYSDNQPGVLIQV YEGER(2658.3374)					
12	heat shock protein, putative	*Ricinus communis*	XP_002518324	FESLVNHLIER(1356.7566) AVITVPAYFNDAQR(1564.8694) GVNPDEAVAMGAAIQGGILR(1939.085) GVNPDEAVAMGAAIQGGILR(1955.0647)	71.08/6.10	66.2/5.56	276/230	4/14	23%
21	heat shock protein 60	*Prunus dulcis*	AAN63805	APGFGENRK(975.5055) MISTSEEIAQVGTISANGER(2093.0415)	57.73/5.26	58.4/5.61	214/123	2/19	37%
46	hypothetical protein OsI_01440 (Peptidyl-prolyl cis-trans isomerase)	*Oryza sativa* Indica Group	EEC70431	TPWLDNR(901.4586)	25.36/8.05	18.16/5.86	86/48	1/7	32%
**Signal transduction**									
30	calreticulin, putative	*Ricinus communis*	XP_002514817	TLVLQYSIR(1092.6418) AREEEEAQR(1117.5186)	49.78/5.81	47.40/6.06	89/74	2/9	14%
44	14-3-3-like protein	*Zea mays*	NP_001105677	DSTLIMQLLR(1189.6669) DSTLIMQLLR(1205.6462) IVSSIEQKEESR(1404.7404) TVDVEELTVEER(1418.7095)	28.87/4.82	31.26/4.96	258/188	4/13	41%
**Proteometabolism**									
3	proteasome subunit beta type 6,9, putative	*Ricinus communis*	XP_002527995	TVIINSEGVTR(1188.6373) SGSAADSQIVSDYVR(1554.7103) YFLHQHTIQLGQPATVK(1981.0228)	24.85/5.17	23.03/5.45	365/316	3/8	50%
15	disulfide isomerase	*Gloeospermum blakeanum*	ACZ95473	SASGNLVQYDGDR(1381.61)	10.92/4.44	62.08/5.01	77/66	1/2	15%
8	Cell division protein ftsH, putative	*Ricinus communis*	XP_002520373	FLEYLDKDR(1198.5884) TPGFSGADLANLLNEAAILAGR (2171.1414) KVDLFENGTIAIVEAVSPELGNR (2471.3274)	75.33/6.43	66.2/5.38	568/446	3/23	34%
38	eukaryotic translation initiation factor 5A	*Gossypium hirsutum*	ADG27839	TYPQQAGTIR(1134.6205) TYPQQAGTIRK(1262.715)	17.40/5.61	14.84/5.63	116/91	2/5	24%
42	Protein kinase domain containing protein, expressed	*Oryza sativa* (japonica cultivar-group)	ABA94760	AAMQQRGR(917.4733) VASNCLATMDNKR(1422.6827) NVIGPQSRTKPAPHLQFR(2046.1355)	80.25/9.98	31.00/6.44	99/0	3/21	28%
**Amino acid metabolism**									
24	phosphoglycerate dehydrogenase	*Gossypium hirsutum*	ACJ11736	EVFESSGGR(967.4564) GGVIDEEALVR(1157.6221) GLGMHVIAHDPYAPADR(1835.8821)	63.78/7.14	60.74/5.85	197/171	3/10	16%
28	Ketol-acid reductoisomerase,chloroplastic	*Pisum sativum*	O82043	DLFHLLPDAFK(1315.6791) GILLGAVHGIVESLFR(1680.9719) GILLGAVHGIVESLFRR(1837.0664) QIGVIGWGSQGPAQAQNLR(1980.0358) EINGAGINSSFGVHQDVDGR(2071.9685)	62.81/6.62	58.4/6.09	366/306	5/14	23%
40	3-isopropylmalate dehydratase, putative	*Ricinus communis*	XP_002530662	NSVATGEIYPLETEVR(1777.9103)	26.91/6.43	25.91/5.14	118/111	1/2	13%
29	AlaT1	*Vitis vinifera*	AAZ43369	EVAEFIGRR(1076.583) GVMQILNTIIR(1257.7366) GVMQILNTIIR(1273.717)	53.66/6.54	47.40/6.14	467/376	5/17	31%
				HYLSLTSGGLGAYSDSR(1783.8708) IIFTNVGNPHALGQKPLTFPR(2320.3147)					
48	glutamine synthetase	*Cucumis melo*	AAX35343	HKDHISAYGEGNER(1612.736)	47.63/8.06	40.49/5.97	133/101	1/9	22%
**Others**									
23	predicted protein	*Populus trichocarpa*	XP_002322147	QLFIDGEWR(1163.5789) QLFIDGEWREPVLK(1729.9249)	54.88/5.25	58.37/5.56	114/108	2/3	5%
33	unknown	*Glycine max*	ACU22776	AAYNNPER(934.4539) NSQQFQALR(1091.5814)	41.17/4.8	48.18/5.00	121/99	2/6	21%
41	Unknown	*Populus trichocarpa×Populus deltoides*	ABK96657	GNELWYGPDR(1206.589) VDFKEPVWFK(1294.7012) GNELWYGPDRVK(1433.7603)	24.36/4.77	26.13/4.85	134/106	3/6	17%
47	hypothetical protein	*Vitis vinifera*	XP_002271	VAGAAADILGAASHYGK (1571.8383)	14.97/6.69	18.16/5.61	129/119	1/2	24%

^a^ Numbering corresponds to the 2-DE gel in Figure 3.

^b^ The number of the predicted protein in NCBInr.

^c^ The matched peptide sequences.

^d^ Molecular mass and p*I* theoretical and experimental.

^e^ Protein score and ion score.

^f^ Number of matched peptides and total searched peptides.

^g^ Percentage of predicated protein sequence with matched sequence.

**Figure 6:**
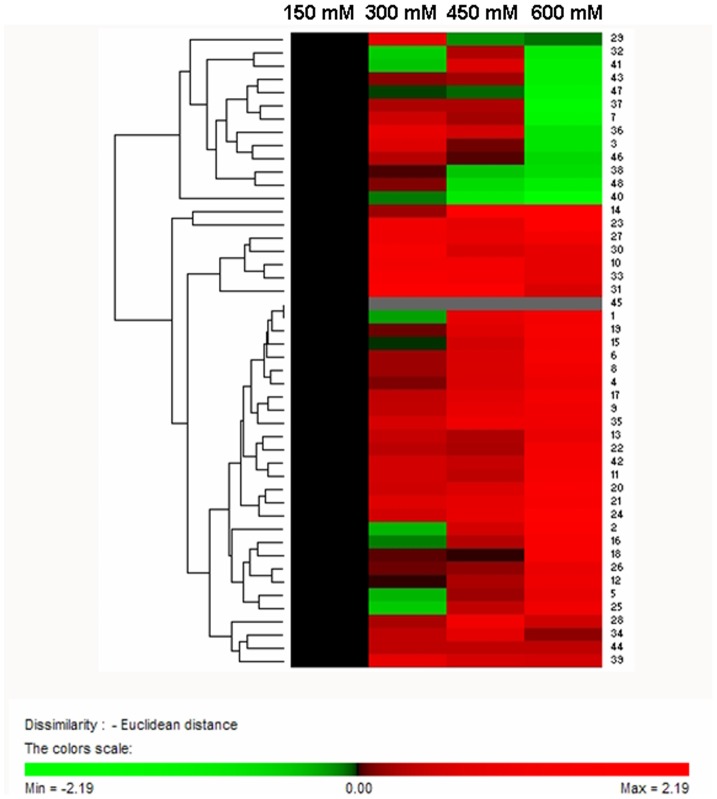
Hierarchical clustering analysis of the 48 differentially expressed proteins from *K. candel* leaves under different salinity levels. The rows represent individual proteins. The proteins that increased and decreased in abundance are indicated in red and green, respectively. Proteins not detected on control gels are indicated in gray. The intensity of the colors increases as the expression differences increase, as shown in the bar at the bottom.

A total of 48 salt-responsive proteins were submitted to a hierarchical clustering to group proteins showing similar expression patterns under salt treatment. Two main clusters were formed (Figure 6): one cluster included 13 proteins whose abundance decreased under 600 mM NaCl treatment compared with the non-saline; the other cluster contained 35 up-regulated proteins. Examples for proteins that were decreased under severe salinity conditions are a chlorophyll a-b binding protein (CAB) 2 (spot no. 7), chlorophyll A/B binding protein (CAB) (spot no. 37), oxygen evolving enhancer protein 2 (OEE2) (spot no. 43), whose abundance increased under 300–450 mM NaCl treatment but decreased under 600 mM NaCl treatment, respectively. These changes in protein abundance may imply that photosynthesis is down-regulated under high salt conditions. From the increased cluster tree, most of the proteins were up-regulated with the increase of salinity. These proteins are mainly involved in carbohydrate metabolism and energy, protein metabolism, and signal transduction. For instance, the proteins involved in carbohydrate metabolism and energy (spot nos. 4, 6, 17, 18, 19, 20, 22, 25, 27, 34 and 39) were increased significantly by salt adaptation treatment compared with control. In addition, this cluster also comprised other salt inducible proteins, such as photosynthesis related proteins (spot nos. 13, 14, 16, 31 and 35) and a detoxifying and antioxidant related enzyme (spot no. 45).

### Immunoblot analysis for DHAR and HSC70

**Figure 7:**
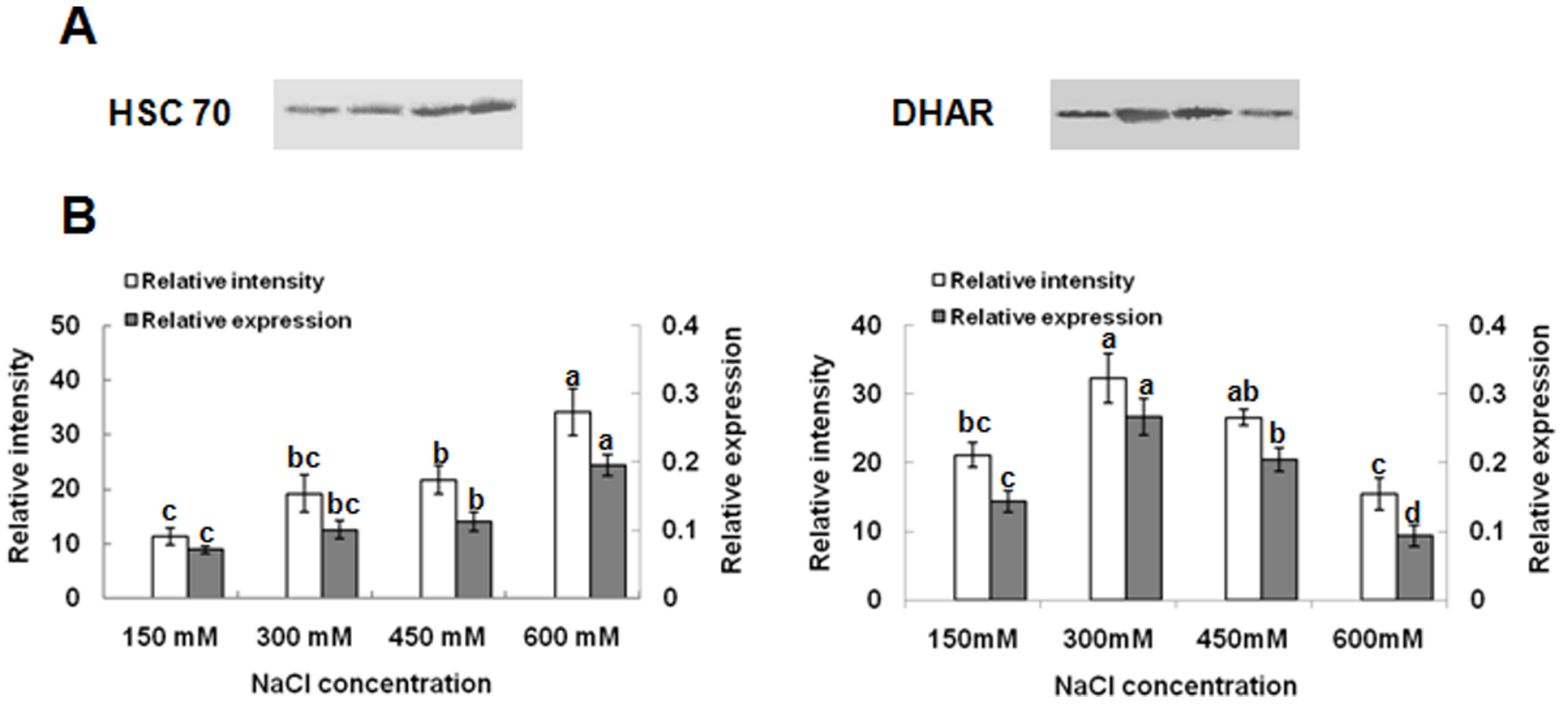
Western blot analysis of HSC 70 and DHAR expression patterns and the relative expressions on protein levels in *K. candel* leaves. (A) Antibodies against HSC 70 and DHAR were used to detect the change of protein levels in leaves in response to salt stress treatment of the plants. 50 µg protein samples were loaded in each lane, separated on 12% SDS-PAGE gel, followed by Western blotting and visualized with DAB as described in Materials and Methods section; (B) Gray analysis of the results by Quantity One software and the relative expression of HSC 70 and DHAR (spots 11 and 36) were shown. Bars represent the means of the relative intensity of the protein stains of three biological replicates of the control and salt-stressed treatments (150, 300, 450 and 600 mM NaCl).

In the current study, the accuracy of 2-DE analysis was further validated by immunoblot analysis. Proteins of *K. candel* leaves were separated by one-dimensional SDS-PAGE, and immunoblot analysis was performed for DHAR and heat shock cognate protein 70 (HSC70) (Figure 7). In agreement with the changes in protein abundance observed by 2-DE, DHAR showed an increased amount in response to 300 and 450 mM NaCl treatment (Figure 7A). HSC70 immunoblot analysis revealed an increase in the amount of cross-reacting polypeptide bands in response to high salinity (Figure 7B).

## Discussion

Although there have been two previous reports about proteins related to salt tolerance in mangrove (*B. gymnorrhiza*), only 3 and 10 proteins were identified, respectively. Tada and Kashimura [36] identified FBP-aldolase, osmotin, and a novel protein in the root. Zhu et al. [37] reported that salt tolerance to 200 mM NaCl was due to increased expression of RuBisCO activase, glutathione transferase, and heat shock proteins (HSPs), which improved the salt tolerance of *B. gymnorrhiza*. A reduction in the abundance of the above proteins was observed after treatment with 500 mM NaCl. Our study has revealed that additional important proteins are related to salt tolerance in the mangrove plant *K. candel*. Forty-eight differentially expressed proteins were identified that are known to be involved in several cellular processes such as photosynthesis, respiratory and energy metabolism, detoxification and antioxidation, as well as signal transduction.

### Proteins involved in photosynthesis

**Figure 8:**
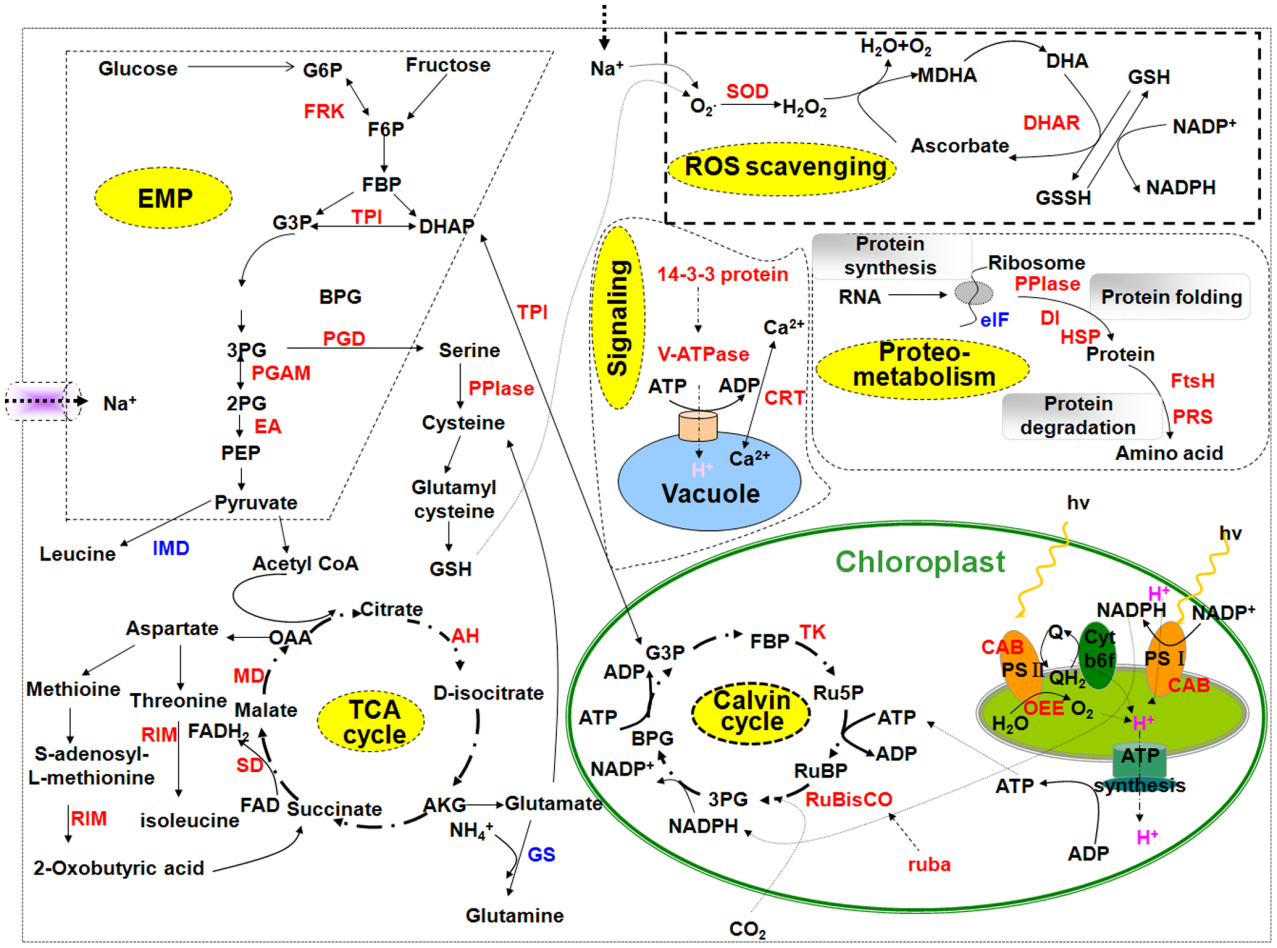
Schematic presentation of a mechanism for salt tolerance in *K. candel*. Most differentially expressed proteins were integrated, and were indicated in red (up-regulated at least under 450 mM NaCl treatment) or blue (down-regulated), respectively. Abbreviations: ADP, adenosine diphosphate; AKG, oxoglutarate; BPG, 1,3-bisphosphoglycerate; cytb6f, cytochrome b6f; DHA, dehydroascrobate; DHAP, dihydroxyacetone phosphate; EA, enolase; eIF, eukaryotic translation initiation factor; F6P, fructose-6-phosphate; FADH_2_, reduced flavin adenine dinucleotide; FtsH, Cell division protein ftsH; G3P, glyceraldehydes-3-phosphate; G6P, glucose-6-phosphate; GS, glutamine synthetase; GSH, reduced glutathione; GSSH, oxidized glutathione; IMD, isopropylmalate dehydratase; MDHA, monodehydroascorbate; MDHAR, MDHA reductase; NADP^+^/NADPH, nicotinamide adenine dinucleotide phosphate; OAA, oxaloacetic acid; PEP, phosphoenolpyruvate; PG, phosphoglycolate; PGD, phosphoglycerate dehydrogenase; PPIase, peptidyl-prolyl cis-trans isomerase; PRS, proteasome; Q, quinone; R5P, ribose-5-phosphate; Ru5P, ribulose-5-phosphate; RuBisCO, ribulose-1,5-bisphosphate carboxylase/oxygenase; RuBP, ribulose-1,5-bisphosphate; RIM, reductoisomerase; TPI, triosephosphate isomerase.

Photosynthesis is sensitive to be affected by salinity among the primary metabolism in high plant [38]. Proteomic analysis revealed that a several proteins associated with photosynthesis was differentially expressed upon salt stress (Figure 8). As shown in Table 1, 11 enzymes involved in photosynthesis were positively identified. Of these, 3 enzymes (spot nos. 7, 37, 43) have important roles for light-dependent reactions; the other 8 enzymes (spot nos. 4, 5, 6, 14, 16, 13, 31, 35) take part in the Calvin cycle. All 11 proteins were up-regulated under 300 mM and 450 mM NaCl stress. For light-dependent reaction related proteins, the expression level of OEE2 (spot no. 43), CAB 2, chloroplastic (spot no. 7) and CAB (spot no. 37) were slightly up-regulated by exposure to 300 mM and 450 mM NaCl but decreased dramatically in response to severe stress (600 mM NaCl). Previous studies reported that OEE, consisting of OEE1, OEE2, and OEE3, play an important role in the light-induced oxidation of water in photosystem II (PSII) of plants [39]. However, these subunits in PSII complex can be easily dissociated under salt stress [39]. Up-regulation of OEE2 has been reported in *Bruguiera gymnorrhiza* in response to salt stress, which suggests that it might be needed to repair the injury of the PSII complex and to maintain the oxygen evolution reaction [36]. CAB 2 (spot no. 7), a component of the light-harvesting complex of photosystem I (PSI), facilitates light absorption and transfer of the excitation energy to reaction centers for the reduction of NADP^+^ to NADPH [40]. As to Calvin cycle related proteins, RuBisCO large subunit (spot nos. 31 and 35), RuBisCO large subunit-binding protein subunit beta (spot no. 13) and RuBisCO subunit binding-protein alpha subunit (spot nos. 14 and 16) were all significantly increased in abundance under all salt treatments. RuBisCO subunit binding-protein alpha subunit is important in RuBisCO complex assembly [41]. RuBisCO large subunit-binding protein subunit beta (ruba) (spot no. 13) is related to RuBisCO activation [42]. The chloroplast-localized protein transketolase (TK) (TargetP [43]; spot nos. 4, 5, and 6), which located in chloroplast by subcellular prediction tool, which is involved in the regeneration phase of the Calvin cycle, was also up-regulated under salt stress. The above results suggested that *K. candel* could withstand up to 450 mM NaCl stress possibly through the up-regulation proteins related to the light-dependent reactions, which in turn provides adequate amounts of energy equivalents necessary for the Calvin cycle and other processes important during salt stress. In addition, the up-regulation of the Calvin cycle produces more photosynthetic products, such as sucrose and starch, to improve plant salt tolerance [44].

### Proteins related to carbohydrate and energy metabolism

Large amounts of energy are needed for the growth and development of *K. candel* under salt stress. This energy is mainly produced through carbohydrate metabolism, such as glycolysis (EMP) and tricarboxylic acid cycle (TCA) [27], [45], [46]. Fifteen differential expressed proteins in *K. candel* leaves under salt stress catalyze several steps in these pathways (Figure 8). 2,3-bisphosphoglycerate-independent phosphoglycerate mutase (PGAM) (spot no. 27) and fructokinase-1 (FRK) (spot no. 32) are enzymes of EMP. PGAM was up-regulated up to 600 mM NaCl stress while FRK reached maximum abundance under 450 mM salt concentration. FRK catalyzes the transfer of a phosphate group from ATP to fructose in glycolysis and is the most important gateway in the control of sugar influx into EMP [47]. Furthermore, three protein spots representing aconitate hydratase (AH) (spot no. 1), succinate dehydrogenase (SD) (spot no. 25), and malate dehydrogenase (MD) (spot no. 34) were found to be up-regulated. The first two enzymes are key enzymes in the TCA cycle. Thus, the increase of FRK under NaCl along with the other enzymes of EMP-TCA in this study would contribute to glucose breakdown for energy generation to cope with salt stress. In addition, mitochondrial F1–ATPase beta subunit (spot nos. 18 and 19), ATP synthase CF1 alpha subunit (spot no. 20), and F1 ATPase (spot no. 22), involving in ATP synthesis [42], were up-regulated under salt stress in this study. ATP, the main source of energy, is indispensable for many metabolism pathways in higher plants. The energy requirements in response to external stress may considerably increase [48]. The up-regulated ATP synthesis indicates that ATP formation is one of the strategies of plants to cope with salt stress. The results above indicated that EMP-TCA activity coupling with ATPase in *K. candel* leaves was enhanced suggesting that the respiratory metabolism in *K. candel* was increased under salt stress. Increased EMP-TCA activity and ATP synthesis imply that salt stress forces the plant to remobilize energy to cope with salt stress [49]. Together with the amplification of the light reactions of photosynthesis, this may insure the continuous generation of ATP and NAD(P)H necessary to mediate the enhanced salt resistance in *K. candel* [35], [50]. Thus, the species seem to derive its ability to improve stress tolerance through the adjustment of its-energy metabolism.

### Proteins associated with detoxification and antioxidation

Vacuolar H^+^-ATPase transports protons across the tonoplast, resulting in the formation of a proton gradient. The resulting gradient provides the driving force for active Na^+^ transport into the vacuole [51], and prevents the cytoplasm from reaching toxic Na^+^ levels. This sequestration of Na^+^ into the vacuole is a crucial and effective strategy for reducing Na^+^ concentration in cytoplasm and regulating cell osmosis in plant [34]. In the present study, both the Na^+^ concentration (Figure 2A) and the expression of vacuolar H^+^-ATPase (spot 2) were increased (Figure 4) and K^+^/Na^+^ ratios (Figure 2B) only decreased slightly with increasing salt content as the NaCl treatment progressed in leaves under salt stress. These results were consistent with the reports of a study by Wang et al. [35]. In the present study, *K. candel* is likely to have an ability to sequester Na^+^ into vacuole and maintain K^+^ homeostasis under NaCl stress. This active Na^+^ efflux requires a H^+^ gradient across the vacuolar membrane generated by stimulating protein expression of the vacuolar H^+^-ATPase [52], suggesting that up regulation of vacuolar H^+^-ATPase might play a vital role in salinity tolerance of *K. candel*.

Salt stress induces reactive oxygen species (ROS) accumulation which may lead to oxidative stress, and high NaCl concentration is harmful to plant cell components [6], [53]. In plants, however, ROS may be considered as signaling molecules to increase antioxidant enzymes for adapting to high salt levels [4]. With further increases in salt levels, the detoxification roles of oxidant-tolerant enzymes may dominate ROS signaling effects, indicating that the plant can use detoxifying and antioxidant enzymes to respond to high salinity [35]. Spot no. 45 showed homology with chloroplast SOD. The function of SOD is to catalyze the conversion of O_2_^−^ to H_2_O_2_ and O_2_ during various stresses, which is deemed to be one of the first lines of defense against free radical damage in plant cells [54]. Accumulation of SOD in response to salt stress has been reported to play a protective role in canola [4] and *S. europaea* [35]. In this study, SOD levels increased in response to salinity, with a more pronounced increase at 450 mM NaCl and 600 mM NaCl (Figure 4). As a result, O_2_^−^ content was not significantly increased at 300 and 450 mM NaCl, and only slightly increased under high salt stress (600 mM NaCl), suggesting that salt-tolerant *K. candel* an increased capacity for scavenging O_2_^−^ (Figure 1D).

Furthermore, we detected an increase in the levels of DHAR1 (spot no. 36) in response to 300 and 450 mM NaCl in our proteomics (Figure 4). This increase was confirmed using Western blot analysis (Figure 7A) esearch with privious ions. The previous report indicated that this protein was up-regulated under salt stress [31]. Increases in DHAR abundance may be a result of H_2_O_2_ removal through the production of ascorbic acid during stress [55]. DHAR is frequently designated as an enzyme to protect against oxidative stress in plants [56]. Previous studies indicated that the antioxidative defense system as a whole was induced during salt stress for scavenging ROS [6], [57]. Mandhania et al. [58] and Yan et al. [59] reported that antioxidant enzymes are up-regulated at the protein level by salt stress in both, wheat and rice. In the present study, H_2_O_2_ content remained low during treatments with up 300 mM to 450 mM NaCl, but increased obviously at 600 mM NaCl (Figure 1C). This is consistent with our findings that SOD and DHAR activities and protein levels increase during exposure of up to 450 mM NaCl (Figure 1A and B). These results support the idea that functionally active antioxidant enzymes, SOD and DHAR, could be used to enhance resistance up to 450 mM salt stress in *K. candel*.

### Chaperones

Salt stress results in protein misfolding or unfolding, which injures plant cells. To avoid these, cells produce molecular chaperones, such as the members of HSPs, which assist protein folding or assembly and prevent irreversible protein aggregation by maintaining native conformations during salt stress [32], [34], [35], [45]. Under adverse conditions, HSPs can protect plants against stress by refolding proteins to reestablish normal protein conformation and maintain cellular homeostasis [60]. Previous studies reported that HSC 70 was implicated in a variety of cellular processes, including the folding of nascent chain polypeptides or the import/translocation of mitochondrial or chloroplast precursor proteins [60], [61]. Another report indicated that HSP 60, a mitochondrial chaperone, plays a vital role in the transport of proteins from the cytoplasm into the mitochondrial matrix and in the refolding of proteins, thus preventing protein aggregation when the mitochondria are subjected to stress [62]. In this study, Non–cell-autonomous heat shock cognate protein 70 (spot no. 9), high molecular weight HSP (spot no. 10), HSC 70 (spot no. 11), HSP, putative (spot no. 12), and HSP 60 (spot no. 21) were identified. These proteins were all up-regulated by exposure to high salinity, suggesting that the proteins play a crucial role in aiding the folding and assembly proteins under salt tolerance in *K. candel* seedlings (Figure 8). These results are similar with the report by Zhu et al. [37] in mangroves plant, *Bruguiera gymnorhiza*. This was further confirmed by an increase in protein abundance in response to salt-treatment as determined by Western blot (Figure 7B).

### Proteins involved in signal transduction

One of the proteins that increased abundantly during salt stress is a 14-3-3-like protein (spot no. 44) (Figure 4). In plants, the 14-3-3 proteins, a highly conserved family, are known to be involved in responses to diverse stresses including salinity [49], [63]. The biological roles of 14-3-3 complexes are in the regulation of primary metabolism, signal transduction, and subcellular and defense reactions [64]. They are also recognized as positive regulators of plasma membrane H^+^-ATPase in the regulation of ion transport and cytoplasmic pH [65], [66]. Moreover, 14-3-3 proteins have been implicated in various signal transduction pathways through controlling the activities of kinases and phosphatases [67], [68], which suggests that 14-3-3 proteins regulate multiple pathways involved in salt stress responses in higher plants [49]. As shown in Figure 4, calreticulin (CRT) (spot no. 30) was up-regulated in response to salt treatment. CRT, one of the most important calcium-binding proteins, is involved in calcium signaling in the endoplasmic reticulum during the stress response in plants [60], [69]–[72]. Therefore, the results indicated that up-regulation of 14-3-3-like proteins and CRT might play roles in signal transduction in *K. candel* under salt stress.

### Proteometabolism

Disulfide isomerase (DI) (spot no. 15) is an enzyme that participates in disulfide bonds formation and breakage between cysteine residues when proteins folding, which was up-regulated in this study (Figure 8) [73], [74]. These reactions will lead to the rearrangement of disulfide bonds in a single protein that exist intra-molecularly. Proteasome, which involves in regulating the particular protein concentration, can degrade unneeded or damaged proteins in plant cells. In the present study, proteasome subunit beta type 6, 9, putative (spot no. 3) was up-regulated by 300 and 450 mM NaCl but down-regulated by 600 mM NaCl, suggesting that degradation of unneeded, damaged, and misfolded proteins by the proteasome pathway was active in plant resistance to salt toxicity under at least 450 mM concentration.

## Conclusions

In the present study, we provided a comprehensive proteome dynamics of the leaves in the woody halophyte *K. candel* under salt stress. Forty eight differentially expressed proteins, showing more than a 2-fold change in abundance, were identified. As a result, we gained new information about proteins in *K. candel* and their role in the stress response. First, proteins involved in light-dependent reactions, Calvin cycle and respiration were up-regulated to improve salt resistance under moderate NaCl treatment (300–450 mM), but some of them were down-regulated under high salt stress (600 mM NaCl). Their function could be to maintain photosynthetic electron flow and to provide energy equivalents necessary for repairs. The second strategy involved the up-regulation of proteins leading to an energy remobilization in *K. candel* under all NaCl treatments. Third, vacuolar H^+^-ATPase played a vital role in Na^+^ detoxification in the plant cell, while up-regulation of SOD and DHAR prevented the accumulation of ROS. Our proteomic data revealed that a series of exquisite biochemical mechanisms for salt tolerance of *K. candel* which enable this species to withstand up to 450 mM NaCl stress. The proteome result was corroborated by immunoblots of the representative proteins and measurements of SOD and DHAR activities, as well as the content determinations of H_2_O_2_, O_2_^−^, Na^+^, and K^+^/Na^+^. It is noteworthy that several novel salt-responsive proteins are identified in this study compared with previous reports in mangrove plants. This study has allowed us to expand our knowledge of the mechanisms by which woody halophytes respond to salinity and may assist in designing and developing more salt-tolerant plants in the future.

## Materials and Methods

### Plant growth conditions

Mature hypocotyls of *Kandelia candel* were obtained from the estuary of Luoyang river (24°58′N, 118°39′E), Quanzhou city, Fujian province, China, which features salinity levels ranging from 8‰ to 20‰. We conducted the study in the experimental zone of the mangrove reserve that obeys the Article 18 of Regulations of the People's Republic of China on Nature Reserves. Similar-sized hypocotyls were chosen for the experiment, which was conducted in a sand culture system under the following conditions: a thermo-period of 30/25°C (day/night), a photoperiod of 16 h/8 h (day/night), light intensity ranging from 1,200 µmol m^−2^ s^−1^ to 1,250 µmol m^−2^ s^−1^, and a relative humidity of 70%. Since *K. candel* is a salt-tolerant plant and naturally grows best in sea water with a salt concentration of approximately 150 mM, the hypocotyls were cultivated in Hoagland solution (Macronutrients: 5 mM Ca(NO_3_)_2_, 5 mM KNO_3_, 2 mM MgSO_4_·7H_2_O, 1 mM KH_2_PO_4_ and 0.1 mM EDTA-Na_2_; Micronutrients: 46 µM H_3_BO_3_, 9 µM MnCl_2_·4H_2_O, 0.3 µM CaSO_4_·5H_2_O, 0.7 µM ZnSO_4_·7H_2_O and 0.08 µM (NH4)_6_Mo_7_O_24_ •4H_2_O) containing 150 mM NaCl in rectangular plastic trays (30 cm×42 cm×14 cm) until four leaves had fully expanded. Subsequently, the seedlings were subjected for 3 d to 150 (control), 300, 450, and 600 mM NaCl in a split plot design with three replicates. The leaves were harvested and kept at −80°C. Three independent biological replicates were prepared for physiological and proteomic analyses.

### Na^+^ and K^+^ ion determination

Leaves of seedlings were weighed, washed in distilled water and dried in a 80°C oven until a constant weight. Dry powder (0.3 g) of each sample was digested in concentrated H_2_SO_4_. Na^+^ and K^+^ content in the leaves were determined by the flame emission method as described by Chuang et al. [75] using a flame photometer (FP 640, Shanghai, China). Five biological replicates were extracted for each treatment.

### Enzyme extraction and activity assay

Leaf samples (1 g) were ground into powder in liquid N_2_. Four volumes of 50 mM potassium phosphate buffer (pH 7.0) containing 1 mM EDTA, 2% (w/v) polyvinylpyrrolidone (PVP), and 0.05% Triton X-100 were added and homogenized following the procedures described by Gossett et al. [76]. Centrifugation at 10,000×g for 15 min at 4°C, the supernatant was applied to measure enzyme activity as follows. SOD activity was estimated using the inhibition of photochemical reduction of nitroblue tetrazolium (NBT) at 560 nm [77]. One unit of SOD activity was defined as 50% inhibition of NBT photoreduction DHAR activity was analyzed by the method of the previous report [78]. The activities of DHAR were expressed by measuring the ascorbic acid in absorbance at 265 nm. Protein content was measured as previously described by Bradford [79].

### Analysis of H_2_O_2_ and O_2_^−^ content

For H_2_O_2_ content, leaf tissues (1 g) were ground in liquid N_2_ and then homogenized in 5 ml cold acetone. After centrifugation at 3,000 g, 4°C for 10 min, the supernatants were used for H_2_O_2_ content assays. According to the method of Patterson et al. [80], H_2_O_2_ content was assayed by analyzing the production of titanium–hydroperoxide complex at 410 nm. To determine the O_2_^−^ content, 1 g of leaf tissue was ground to a fine powder in liquid nitrogen and then homogenized in 5 mL of of extraction buffer (pH 7.8) containing 50 mM sodium phosphate, 5 mM EDTA, and 1% (w/v) PVP. After centrifugation at 10,000 g, 4°C for 20 min, the supernatants were collected and the O_2_^−^ content was detected O_2_^−^ contents was assayed using the oxidation of hydroxylamine at 530 nm as described by Wang and Luo [81].

### Protein extraction

Total leaf proteins were extracted using a phenol extraction procedure [82], [83]. Leaf tissues (3 g) of each sample were ground with liquid nitrogen with mortar and pestle, suspended in 12 mL of cooled extraction buffer containing 50 mM L-ascorbic acid, 100 mM KCl, 50 mM disodium tetraborate decahydrate, 0.5% (v/v) Triton X-100, 2% (v/v) β-mercaptoethanol, 2% PVP, 1 mM phenylmethylsulfonyl fluoride and 100 mM Tris-HCl, pH 8.0. An equal volume of ice-cold Tris-HCl-saturated phenol (pH 8.0) was added to the solution, vortexed and the suspension re-homogenized on ice prior to centrifugation at 5,500×g for 10 min at 4°C. Subsequently, the phenolic phase was transferred to second tube, six-fold 100 mM ammonium acetate/methanol added for an overnight incubation at −20°C, followed by centrifugation at 4°C, 20,000×g for 20 min. The pellet was rinsed with cold methanol and washed with acetone containing 0.07% (v/v) β-mercaptoethanol twice. The pellet was collected and lyophilized at −20°C. Protein concentration was determined by the Bradford assay with bovine serum albumin as the standard [79].

### Two-dimensional gel electrophoresis of proteins

Two-dimensional gel electrophoresis was undertaken according to the method of Liang et al. [84] and Liu et al. [85] with the Ettan IPGphor system (GE Healthcare, Little Chalfont, UK). The leaf protein (1,500 µg) was loaded onto IPG strips (24 cm, linear gradient pH 4–7; GE Healthcare) in a rehydration tray for 12 h. Isoelectric focusing (IEF) was performed under the following conditions: 200 V for 1 h, 500 V for 1 h, 1 kV for 1 h, gradient to 8 kV for 0.5 h, and, finally, 8 kV for a total of 48,000 Vhs at 20°C. After IEF, the IPG strips were equilibrated in equilibration buffer [50 mM Tris–HCl, pH 8.8, 6 M urea, 30% (v/v) glycerol, and 2% (w/v) SDS] containing 1% (w/v) dithiothreitol for 15 min and then in equilibration buffer containing 2.5% (w/v) iodoacetamide for 15 min. The strips were transferred onto vertical 12% SDS-PAGE gels, and proteins separated at 10 mA/gel for 30 min, followed by 30 mA/gel over night. After electrophoresis, gels were visualized using Coomassie brilliant blue (CBB) R-250 staining. The CBB-stained 2-D gels were scanned using a UMAX Power Look 2100×L scanner (Maxium Technologies, Taipei, China) and analyzed using Imagemaster™ 2D Platinum software version 5.0 (GE Healthcare). Relative comparison of the intensity abundance between control and NaCl-treated (three replicate samples for each group) was performed using the Student's test. The protein spots with distinct differences were regarded to have at least a 2.0-fold amount of change.

### In-gel tryptic digestion of proteins and MALDI-TOF-TOF/MS analysis

Protein spots were manually excised from preparative CBB-stained gels and digested with sequencing-grade modified trypsin (Promega, Madison, WI, USA) following the manufacturer's protocol, then incubated at 37°C for 12 h. Tryptic peptides were redissolved in 0.8 µl of matrix solution [α-cyano-4-hydroxycinnamic acid (Sigma, St. Louis, MO, USA) in 0.1% trifluoroacetic acid, 50% acetonitrile] before application to the MALDI plate.

After air dying, samples were analyzed using a 4700 MALDI-TOF-TOF Proteomics Analyzer (Applied Bio-systems, Foster City, CA, USA). Combined MS and MS/MS spectra were submitted to MASCOT (V2.1, Matrix Science, London, UK) by GPS Explorer software (V3.6, Applied Biosystems). Database searches were used the following parameters: NCBInr database (release date: 2010.07.01); taxonomy of green plant; trypsin digest with one missing cleavage; no fixed modifications; MS tolerance of 100 ppm; MS/MS tolerance of 0.6 Da; and possible oxidation of methionine. MASCOT protein scores (based on combined MS and MS/MS spectra) greater than 75 were considered statistically significant (P≤0.05). The individual MS/MS spectrum with the most statistically significant (confidence interval of 95%) ion score (based on MS/MS spectra) was accepted.

### Hierarchical cluster analyses

The expression profiles of differential proteins were analyzed through two-way hierarchical clustering according to the PermutMatrix software (Figure 6). Rows were mean centered, and Euclidean distance and Average Linkage were used for data aggregation.

### Immunoblot analysis

Protein samples (50 µg/lane) were separated using 12% one-dimensional SDS-PAGE gel electrophoresis, transferred onto nitrocellulose membranes, and incubated at room temperature for 2 h with rabbit polyclonal antibodies raised against either DHAR or HSC70 proteins (Agrisera, Sweden) at 1∶5,000 dilution. After washing three times with TBST buffer (0.01 M TBS, 0.1% Tween-20, pH 7.6), the membranes were exposed for 2 h at room temperature to horseradish peroxidase-conjugated goat anti-rabbit IgG at 1∶300 dilution. Positive signals were visualized with 3, 3′-diaminobenzidine.

### Statistical analysis

Data from repeated measurements are shown as mean ± SD. Comparison of differences among the groups was carried out using Student's test. Significance was defined as P≤0.05.
